# Advances in micropropagation, somatic embryogenesis, somatic hybridizations, genetic transformation and cryopreservation for *Passiflora* improvement

**DOI:** 10.1186/s13007-023-01030-0

**Published:** 2023-05-25

**Authors:** Mohammad Aqa Mohammadi, Myat Hnin Wai, Hafiz Muhammad Rizwan, Abdul Qahar Qarluq, Mengjie Xu, Lulu Wang, Yan Cheng, Mohammad Aslam, Ping Zheng, Xiaomei Wang, Wenbin Zhang, Yuan Qin

**Affiliations:** 1 Fujian Provincial Key Laboratory of Haixia Applied Plant Systems Biology, State Key Laboratory of Ecological Pest Control for Fujian and Taiwan Crops, College of Horticulture, College of Life Sciences, College of Agriculture, Fujian Agriculture and Forestry University, Fuzhou, 350002 China; 2 State Key Laboratory for Conservation and Utilization of Subtropical Agro-Bioresources, Guangxi Key Lab of Sugarcane Biology, College of Agriculture, Guangxi University, Nanning, 530004 China; 3 Pingtan Institute of Science and Technology, Fujian Agriculture and Forestry University, Fuzhou, 350002 China; 4 College of Agriculture, Alberoni University, Kapisa, 1254 Afghanistan; 5 Institute of Advanced Study, Shenzhen University, Shenzhen, 518060 China; 6 Institute of Horticultural Research, Nanning Investigation Station of South Subtropical Fruit Trees, Guangxi Academy of Agricultural Sciences, Ministry of Agriculture, Nanning, 530007 China; 7 Xinluo Breeding Center for Excellent Germplasms, Longyan, 361000 China

**Keywords:** Cryopreservation, Genetic transformation, Organogenesis, *Passiflora*, Tissue culture

## Abstract

Passion fruit is an essential commercial plant in the tropics and subtropics, which has lately seen a rise in demand for high-quality fruits and large-scale production. Generally, different species of passion fruit (*Passiflora* sp.) are propagated by sexual reproduction. However, asexual reproduction, such as stem cuttings, grafting, or tissue culture, is also available and advantageous in many instances. Recent research on passion fruit has concentrated on improving and establishing methodologies for embryogenesis, clonal proliferation via (somatic embryos), homozygote regeneration (by anther culture), germplasm preservation (via cryopreservation), and genetic transformation. These developments have resulted in potentially new directions for asexual propagation. Even though effective embryo culture and cryogenics are now available, however the limited frequency of embryogenic callus transformation to ex-vitro seedlings still restricts the substantial clonal replication of passion fruit. Here, in this review the advancement related to biotechnological approaches and the current understanding of Passiflora tissue culture. In vitro culture, organogenesis, cryopreservation, breeding, and productivity of Passiflora will significantly improve with novel propagation approaches, which could be applied to a wider range of germplasm.

## Highlights

- The availability of diverse genetic resources is essential for passion fruit genetic improvements;
- Tissue culture, somatic embryogenesis, genetic transformation and somatic hybridization are essential for cultivar improvement;
- Preservation and cryopreservation are ideal means for passion fruit long time preservation;
- This review provides biotechnological tools and a current understanding of Passiflora spp.
- Prospects proposed for further investigation in the field.

## Introduction

**Fig. 1:**
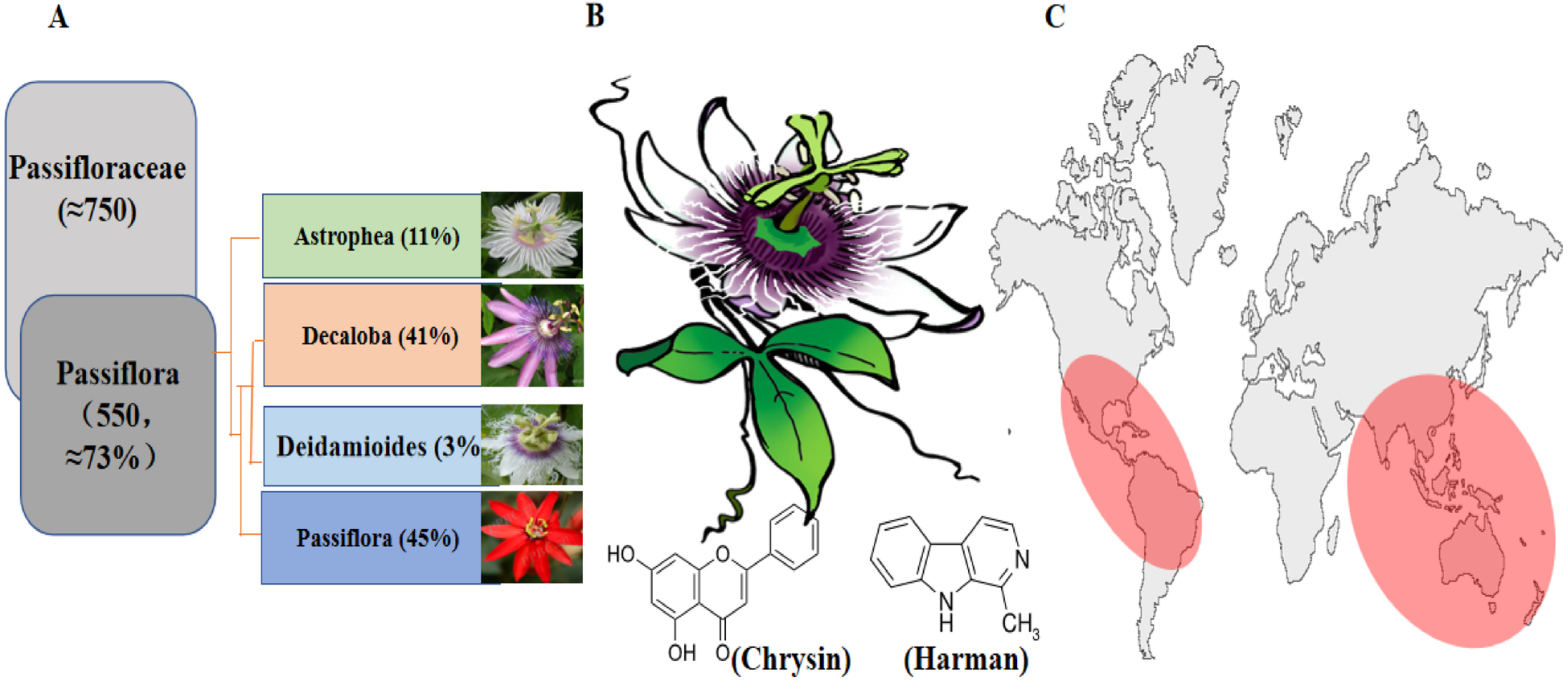
Passion fruit family, species, commercially essential chemicals, and distribution. **A** Indicating the percentage of passion fruit species in the Passifloraceae family and *Passiflora* genera. **B** Flower and leaves of *Passiflora* contain commercially important flavones, chrysin, and an alkaloid (Harman) found in many species of *Passiflora*. **C** The red circles represent the main distribution of *Passiflora* in north, central, and South America, Asia, and Australia

The Passifloraceae family includes the passion fruit (*Passiflora edulis*); the number of species exceeds 500, most of which produce fruits for human consumption and industrial processing. It contains flowers with exceptional beauty and ornamental potential, and phytoconstituents in various parts of the plant are used for potential medical purposes. Many *Passiflora* species are found in tropical and subtropical regions, including Bolivia, Brazil, Colombia, Ecuador, Paraguay, and Peru. Moreover, native species of *Passiflora* are also reported from the USA to Argentina, other than Asia, Australia, and China (Fig. 1) [1–3].

Various passion fruit varieties are commercially developed and serve as a source of revenue and employment in urban and rural areas worldwide [4, 5]. Colombia and Brazil are conventionally major countries developing passion fruit and showing considerable social and economic significance, serving as an alternative crop for family-based agriculture [6, 7]. The fruits and derivatives of *Passiflora* sp. are widely used in pharmaceutical industries [8–10]. The anxiolytics and sedative attributes of *Passiflora* sp. are widely applied in traditional medicine globally. They are also utilized in the food, pharmaceutical, and cosmetic industries. Besides, passion fruit is used as an ornamental plant due to its attractive flowers that come in various forms, sizes, and colors with a unique smell [11, 12]. While a few species, such as *P. edulis* Sims and *P. laurifolia* L., are mainly cultivated for their edible fruits. Others, including *P. morifolia* Mast., *P. suberosa* litoralis (Kunth) K. Porter-Utley, and *P. palmeri* var. sublanceolata Killip, are grown for their unique and spectacular flowers. The shape of its beautiful flowers, which early Christian missionaries to South America characterized as the symbol of Christ’s passion. The edible passion fruit, *P. edulis* Sims, is found in tropical and subtropical latitudes (despite the occasional reports of species in Australia, China, India, and the Pacific island countries) [13, 14]. It has a flavor and taste that is both delicious and unique. The *P. edulis* f. *flavicarpa* cultivar, also called sour passion fruit, accounts for most commercial supply worldwide [15]. Its fruit diameter is about 8–10 cm in size and round in its shape, with a yellowish-green peel at the maturity stage and seeds coated by a gelatinous yellow pulp, a strong aroma, and a sweet-acid taste [16].

## The genus Passiflora’s taxonomy and biodiversity

The Passifloraceae family is thought to have around 700 species. These pronounced variations occur due to taxonomic equivocation, the use of synonyms, and the identification of new species [15, 17]. With estimates ranging from 18 to 23 genera, the number of genera is also debatable. However, apart from taxonomic inconsistencies, the *Passiflora* main genus is undeniably diverse [18, 19]. This mostly tropical genus contains over 500 species spread across five continents, the Pacific Ocean islands, Galapagos Islands, and Brazil [20]. With some species almost endangered or on the verge of extinction, Brazil is the most significant producer and consumer of passionfruit in the world and is regarded as a unique center of the Passiflora variety [21]. Despite the country's enormous local biodiversity, *P. edulis* dominate many commercial gardens [22–24]. On a much smaller level, other species are also grown for their medicinal, flavor, and ornamental properties, for example, *P. nitida* Kunth and *P. cincinnata* Mast [25, 26]. Another phylogenetic controversy regarding the *Passiflora* genus involves the classification of *P. edulis* Sims, and *P. incarnata* L. are synonyms due to phenotypic and microscopic homologies*.* According to Miroddi et al. [27], *P. edulis* species is primarily cultivated for food purposes, and some researchers comment on their pharmacological effects on the central nervous system. They showed that at fewer concentrations, the aerial part of *P.* edulis flavicarpa was anxiolytic, while at high doses, it was sedative [28, 29]. Ozarowski et al. [30] described a summary of essential identification factors, including morphological and physicochemical attributes, that enable the divergence of *P. alata* from *P. incarnata* to lessen the uncertainty between these two species and subsequent selection of the wrong plants (resulting in contradictory pharmacological reports) [30]. Despite their claims, there is still confusion between these two comparable plants. To reduce misidentification issues with *Passiflora* spp. and ensure the authenticity of their related cosmetics and medicinal herbs, the use of pharmaco-botanical methods, which identify morphological and anatomical characteristics, has been proposed as a viable methodology for differentiating similar species [31]. Moreover, [32] research on the volatile components of nine *Passiflora* sp. generated on Madeira Island in Portugal suggested that volatile metabolomic profiling is an effective algorithm for characterizing and differentiating the passion fruit species and varieties, identifying their geographic origins [33].

**Fig. 2:**
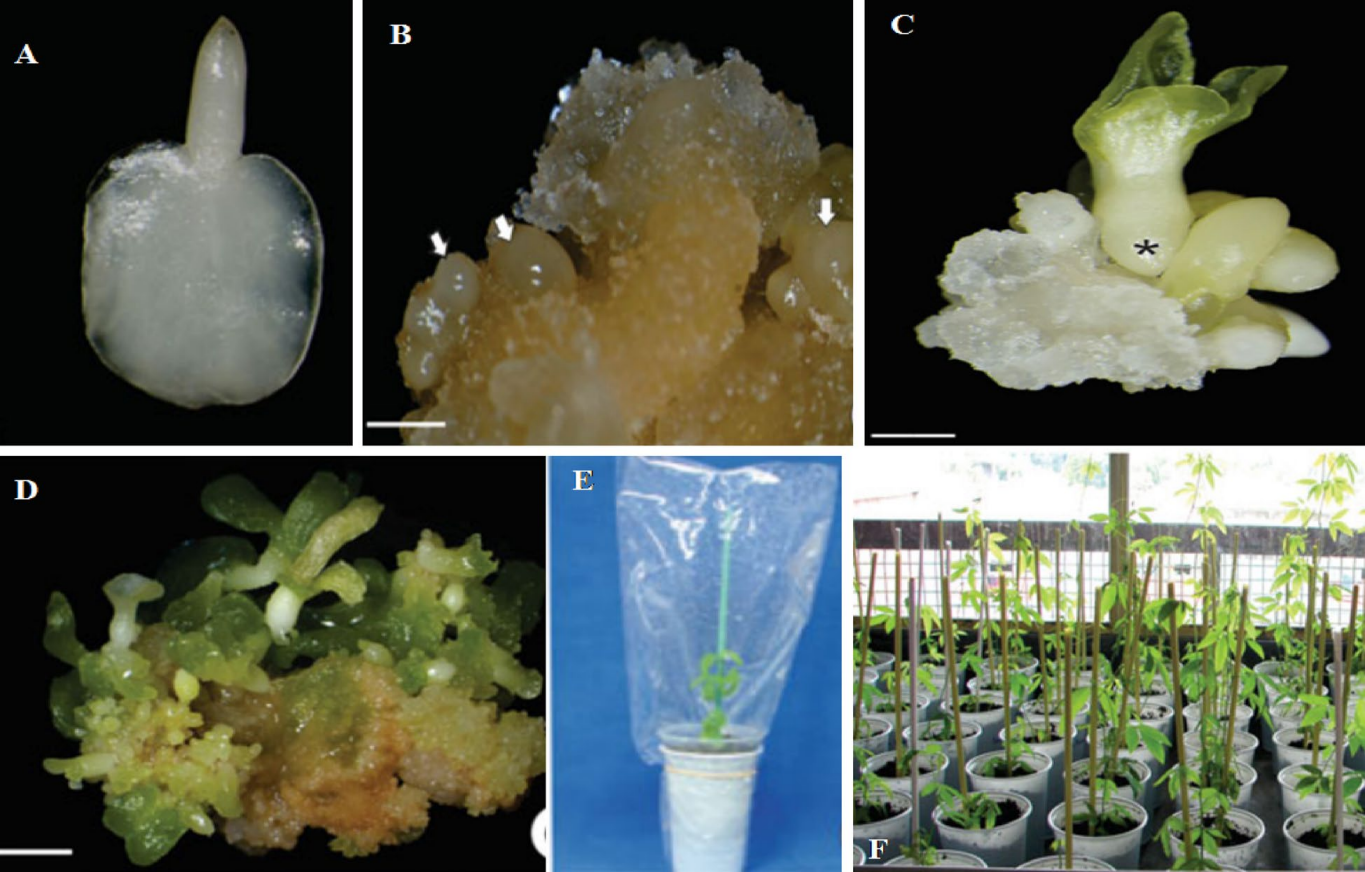
Somatic embryo induction procedure from *P. cincinnata.* **A** Zygotic embryo excised from seed explants. **B** Somatic embryo induced after 30 days in induction medium. **C** Somatic embryo at the cotyledonary stage. **D** Multiple seedlings regeneration from a cotyledonary embryo in a maturation medium. **E** Acclimatization of regenerated plant in a plastic container. **F** Acclimatized regenerated plant grown in the greenhouse the photos **A**–**F** adopted from [38]

Later, the biomarkers were used in the genetic analysis of *Passiflora* spp. to research the diversity of wild *Passiflora* species. Inter-simple sequence repeat (ISSR) markers were employed to evaluate the sweet, purple, and yellow passion fruit accessions genotype. Forty five accessions were examined using 18 ISSR primers [34]. The average number of polymorphic loci per primer was 12.4, ranging from 4 to 22 [34]. The increasing use of molecular markers (particularly co-dominant markers) has increased the availability of knowledge from population-based studies. It will undeniably contribute to further genetic research on the genus and improve current genetic resources [35–37] (Fig. 2).

## Tissue culture studies of *Passiflora* species

### Micropropagation

Several tissue culture and biotechnological methods have been implemented for the *Passiflora* genus due to the identified commercial significance of wild and commercial passion fruit species [39]. Micropropagation investigations in *Passiflora* sp. were initiated in 1966 with the culture of nodal fragments of *P. caerulea*, a passion fruit species with ornamental properties [40]. Since then, many types of research outlining in vitro strategies for economic and wild species with edible, ornamental, and pharmaceutical benefits have been reported [41–43].

Vegetative parts like leaves, stems, nodes, and roots have been used frequently for micropropagation for passion fruit. Passion fruit micropropagation can easily overcome several limitations, such as the lack of uniform healthy planting material for large-scale cultivation. Several studies have been implemented on passion fruit micropropagation to develop a more efficient and effective methodology (Table 1). In these studies (Table 1), different varieties and explants types of passion fruit were studied with varying plant growth regulators medium compositions. The most common medium that has been used was Murushige and Skoog (MS) basal medium [44] or modified MS salts with B5 vitamins [45] (MSM) medium to provide nutrients for plant growth (Table 1).

**Table 1:** Tissue culture for the organogenesis of passion fruit from different explant types and the clonal propagation of passion fruit via somatic embryo

Species name	Explants source	Culture media and optimum PGR combination for shoot induction	Regeneration system	Elongation/prolifferation	PGR for root induction	References
*P.edulis* f. edulis* *P. edulis* f. *flavicarpa*	Leaf disks	MS + 8.88 μΜ BAP	Organogenesis	–	–	[5]
*P. cincinnata* Mast	Zygotic embryo	MS salts + modified B5 vitamins + 0.01% Inositol + 16.22 μΜ2,4-D + 4.44 μΜ BAP	Embryogenic callus, somatic embryo	MS salts + modified B5 vitamins + 0.01% Inositol + 1.5 g/L AC	MS salts + modified B5 vitamins + 0.01% Inositol + 1.5 g/L AC + 1.4 μΜ GA*3*	[38]
*P. cristalina*	Leaf disks, hypocotyls, cotyledon, root segment and endosperm	MS + 8.88 μΜ BAP (leaf and endosperm), MS + 4.44 μΜ BAP (hypocotyls, cotyledon) MS + 4.65 μΜ KN	Organogenesis	–	–	[39]
*P. edulis*	Bud segment	MS + 4.44 μΜ BAP, MS + 1.0 mg mTR; *myo*-Inositol & 170 mg/L NH_2_PO_4_	Organogenesis	–	0.54 μΜ NAA	[41]
*P. edulis* Sims	Internode	MS + 6.66 μΜ BAP + 5.37 μΜ NAA	Organogenesis	–	9.84 μΜ IBA	[42]
*P. cincinnata* Mast	Anther	MS + 9.1 μΜ 2,4-D/18.2 μΜ 2,4-D + 4.44 μΜ BAP	Somatic embryogenesis	–	–	[57]
*P. suberosa* L.	Leaf disks, nodal and intermodal segments	MSM + 44.44 μΜ BAP	Organogenesis	–	1/2MSM	[58]
*P. edulis* f. edulis* *P. edulis* f. *flavicarpa*	Leaf disks	MS + 6.66 μΜ BAP; 1.11 μΜ TDZ; 23.5 μM AgNO_3_	Organogenesis	MSM + coconut water; MSM + GA_3_	–	[60]
*P. alata*	Leaf disks, hypocotyls segment	MS + 2.27 μΜ TDZ + 23.5 μΜ AgNO_3_		MSM + GA_3_	½ MSM	[59]
*P. edulis* Sims	Endosperm	MS + 4.5 μΜ BAP and 9.0 μΜ TDZ; 100 mg/L *myo*-Inositol	Organogenesis	MS	–	[61]
*P. suberosa* L.	Root segments	9.0 μΜ BAP	Organogenesis	1/2MS + 1.5% sucrose	–	[62]
*P. edmundui*, *P. rubra*, *P. alata*, *P. gibertii*, and *P. phohlii*	Pollen grain	SM + 30% sucrose	Pollen tube germination			[63]
*P. miniata* Mast.	Zygotic embryo	MS + 0.01% Inositol + 3.33 μΜ BAP + 2.27 μΜTDZ and 2.32 μΜ KIN	Embryogenic callus	MS + 3.33 μΜ BAP	MS salts	[64]
*P. alata* Curtis, *P. crenata* Feuillet & Cremers, *P. edulis* Sims, *P. foetida* L., and *P. gibertii* N.E. Brown	Zygotic embryo	MS salts + B5 vitamins + 0.01% Inositol + 4.5 μΜBAP + 13.6/18.11 μΜ 2,4-D	Callus, somatic embryo	–	1/2 MS-free hormones	[65]
*P. cincinnata* Mast	Zygotic embryo	MS + 0.01% Inositol, 18.11 µM 2,4-D + 4.5 μΜ BAP	Embryogenic callus, somatic embryo	MS + activated charcoal	MS Free PGRs medium	[66]
*P. miniata* Vanderpl, *P. speciosa* Gardn	Imature zygotic embryo	MS salts + B5 vitamins + 0.01% Inositol	Embryogenic callus, somatic embryo	–	MS salts + B5 vitamins + 0.1% Inositol + 3.0% Activated charcoal	[67]
*P. alat*	Hypocotyl	MS + 4.44 μΜ BAP; 23.95 μΜ AgNO_3_	Organogenesis	–	–	[74]
*P. cincinnata *Mast	Anther	MS salt + B5 vitamins free hormones; 100 mg/L *myo*-Inositol	Somatic embryogenesis	–	–	[76]
*P. alata*	Leaf disks, hypocotyl	MS + 2.27 μΜ TDZ; MS + 4.43 μM BAP + 2.27 μΜ TDZ, 23.5 µM AgNO_3_	Organogenesis	MSM + GA_3_	1/2MSM	[59]
*P. edulis* Sims	Mature zygotic embryo	MS salts + B5 vitamins + 0.01% Inositol + 72.4 μΜ 2,4-D	Embryogenic callus, somatic embryo	MS + 4.5 μΜ BAP	MS + I = IAA-Asp	[112]

Among the known phytohormones, auxins and cytokinins play a significant role in plant tissue culture. For passion fruit, cytokinins are best known to stimulate cell division and axillary bud proliferation [46–48]. As a cytokinin in MS basal medium, the addition of BAP is essential for the organogenesis regeneration of plants from the shoot and root apices of passion fruit [49, 50], besides TDZ also reported to regenerate seedlings from root explants [43]. Several researchers have investigated in vitro rooting induction and the development of passion fruit using different concentrations of rooting medium. MS basal solidified medium, enriched with 9.84 μΜ Indole-6-butyric acid (IBA) [42, 51] and 0.54 μΜ naphthaleneacetic acid (NAA) [11, 41] led to root induction. Therefore, the above results suggested that different concentrations of auxins could induce the rooting of passion fruit in vitro. The selection of the best concentration and the most cost-effective rooting condition should be optimized according to the requirement and available facilities.

Researchers have significantly enhanced the performance of other crops through plant genetics and breeding, such as pineapple [52]. However, conventional and molecular breeding methods for passion fruit are not very helpful in producing improved varieties. Therefore, the propagation of selected varieties via tissue culture seems to be an attractive alternative to meet the demand for high-quality planting material [53, 54]. Passion fruit tissue culture has not been extensively studied due to technical difficulties during indirect somatic regeneration. Several developed protocols have optimized the different regeneration stages during development. Organogenesis is the most common morphogenic pathway reported in *Passiflora* tissue culture protocols and has been described using various explants types.

The genotype appears to be more important than growth regulator composition concentration, and the selection of initial explants for both direct and indirect organogenesis systems from different organs of the plant, including leaf, hypocotyl, nodal segment, root, and meristematic tissues, are used as explant [41, 55, 56].

As stated above, organogenesis is the most common morphogenetic pathway for regeneration in *Passiflora* micropropagation systems. However, a recent study showed that regeneration via embryogenesis is possible from the immature embryo of a wild passion fruit [57]. The MS or MSM medium has been employed with some modification for passion fruit in vitro regeneration.

On the other hand, supplementation with growth regulators varies based on the morphogenetic pathways to be activated. BAP at various concentrations ranging from 2.22 to 8.88 µM is often used to induce multiple shoots via *in-vitro* organogenesis in passion fruit, whereas thidiazuron (TDZ) 1.11–2.33 µM alone or in combination with BAP has also been reported [58–60]. For in-vitro regeneration of *P. edulis* Sims from the mature endosperm, a comparison of three cytokinins—BAP, TDZ, and KIN—found that TDZ was more effective than the others at 4.5 and 9.0 µM [61]. In vitro regeneration of *P. suberosa* was achieved using nodal segments along with internodes [58], root segment [62], in vitro pollen grain germination [63] in the BK and MS media with various concentrations of sucrose, and nodal segments in MS or MSM medium supplanted with NAA, picloram, and 2,4-dichlorophenoxyacetic acid (2,4-D) [58].

### Somatic embryogenesis

For several Passiflora species, somatic embryogenesis from mature and immature zygotic embryos in MS or MSM basal medium with various combinations of 2,4-D (8.8–72.4 μM), and 4.5 μM BAP has been optimized [64] (Table 1). Although zygotic embryos from *P. suberosa* were cultivated under identical conditions as *P. cincinnata*, only adventitious bud development was observed [65]. A regeneration mechanism was reported for *P. cincinnata*, an Amazonian species with significant horticultural potential, via organogenesis from zygotic embryos in a modified MS medium supplemented with different concentrations of 2,4-D and BAP [65]. An average of 40.00 shoots per explant were regenerated directly and indirectly from the callus in the presence of 3.33 μM BAP, 2.25 μM TDZ, and KIN [43]. de Faria et al. [39] recently highlighted *P. cristalina*, another Amazonian species with horticultural potential, for its remarkable responsiveness. When hypocotyl segments and zygotic embryos were cultivated in MS media supplemented with 18.11 µM 2,4-D and 4.43 μM BAP, the authors obtained the maximum mean number of shoots [66]. *P. cincinnata* has demonstrated remarkable in-vitro performance with high regeneration frequency [38]. Somatic embryogenesis systems in *P. cincinnata* Mast. have been established for this species from mature zygotic embryos (Table 1) [66]. However, only a reproducible protocol for zygotic embryos from somatic embryogenesis was obtained in an MS medium fortified activated charcoal and various concentration of 2,4-D µM combined with 4.5 µM BAP [66]. Somatic embryogenesis was induced in *P. cincinnata* using zygotic embryos cultivated in a medium with a high auxin/cytokinin balance (18.1 μM 2,4-D and 4.5 μM BAP). Additional authors have reported effective induction of somatic embryos for other Passiflora species, such *P. miniata*, using a similar technique, *P. alata*, and *P. crenata* [65]. When zygotic embryos were cultivated under identical conditions, only adventitious bud development was seen in *P. suberosa* [65]. Organogenesis from zygotic embryos allowed scientists to examine the regeneration mechanism in *P. miniata* [67]. With an average of 40.0 shoots per explant, regeneration occurred directly and indirectly from the callus, mainly in the presence of 3.32 μM BAP [61]. When *P. cristalina* was grown in MS medium combined with 4.43 μM BAP, the most significant mean of shoots was achieved from hypocotyl segments [39].

Moreover, the embryogenic capacity of Passiflora species has received far less attention. Anthony et al. [68] developed *P. gibertii* somatic embryogenic cell suspensions from leaf protoplast. Da silva et al. [57] outlined how altered *P. cincinnata* anther produced somatic embryos indirectly when the medium contained 18.1 μM 2,4-D and 4.5 μM BAP. Several scientists reported on the induction of indirect somatic embryogenesis from mature zygotic embryos of *P. cincinnata* and *P. edulis* in response to 2,4-D and BAP combinations [57, 59, 66]. The histological and ultrastructural events connected with the embryogenic process were also studied to elucidate the components involved in forming embryogenesis competence and identify the cells and tissues involved in this process [38, 69]. Somatic embryo induction, plantlet regeneration and acclimatation process from zygotic embryo shown in the (Fig. 2).

## *Agrobacterium*-mediated genetic transformation

A key tactic for creating disease-resistant plants is genetic transformation. The main factors limiting the cultivation of passion fruit are the woodiness of the fruit (caused by the passion fruit woodiness virus (CABMV), fusarium wilt (caused by *F. oxysporum* f. sp. *passiflorae*), and bacterial blight (caused by *Xanthomonas axonopodis* pv. *passiflorae*) [70]. The genetic transformation of passion fruit was first characterized by Manders et al. [114]. Genetic transformation is a critical tool for achieving disease-resistant plants of the commercial species of *P. edulis* and *P. alata* [71–74]. Passion fruit genetic transformation studies are currently in their initial phases. Disease-resistant species were generated using the technological tools which have already been reported [38, 73]. Correa et al. [74] adopted *A. tumefaciens*-mediated transformations to generate transgenic *P. alata* genotypes with a cowpea aphid-borne mosaic virus (CAMV)-derived coat protein gene fragment. Despite published findings of *Passiflora* genus genetic transformation, it is far from usual, especially for wild species [57]. According to Alfenas et al. [71], a Brazilian isolate of CABMV produced transgenic yellow passion fruit plants that expressed an untranslated RNA that represented two-thirds of the replicase (NIb, nuclear inclusion b) cistron and one-third of the adjacent coat protein (CP) cistron (Table 2). After numerous failed selfing cycles, one transgenic event named TE5-10-15J was found. It was immune to the three viral isolates tested, showed no symptoms after being artificially inoculated, and tested negative by ELISA. From this plant, cuttings were taken, and the cuttings’ offspring underwent inoculation with four additional virus isolates, with the resulting plants demonstrating equal levels of resistance to each. Similar results were presented by Trevisan et al. [72]. *A. tumefaciens* strain EHA105 was transformed after the full-length coat protein gene (CP) cistron from a severe CABMV isolate was cloned into the pCAMBIA 2300 binary vector (Table 2). Transgenic plants that carried the CABMV CP were immune to the disease. R1, R2, and R3 generations of these seedlings were self-pollinated—were acquired and spread. Transformation efficiency for *P. edulis* was reported to be 0.11 to 0.21% [72] for the passion fruit woody virus (PWV) gene and 0.19 to 0.67% for the CABMV coat protein gene [73] when leaf disks were used as explants for *A. tumefaciens*-mediated transformation. The development of protocols that utilize other regeneration pathways in addition to organogenesis is necessary to increase transformation efficiency. The anti-apoptotic gene (p35) from a baculovirus was inserted into the genome of passion fruit using a biolistics technique [70]. Inoculations of the CABMV, the bacteria *X. axonopodis* pv. passiflorae, and the herbicide glufosinate were undertaken on regenerated plants harboring the p35 gene (Table 2). Some p35+ plants had enhanced herbicide tolerance and *X. axonopodis* pv—passiflorae resistance, as proven by the decline in lesion size compared to non-transgenic counterparts; no plant exhibited CABMV resistance [70].

**Table 2:** *Agrobacterium*-mediated genetic transformation in *Passiflora* spp.

Plant species	Explants	Transformation method	Gene transferred	Transformation efficiency	Selectable marker	References
*P. edulis* f. edulis* *P. edulis* f. *flavicarpa*	Leaf disks	*A. tumefaciens*	GusA gene	0.67	pCAMBIA 1301 contains a hygromycin phosphotransferase (*hpt*) gene	[5]
*Passiflora edulis* f. *flavicarpa* Deg	Hypocotyls segment	Gene bombardment	pEPT8p35 and pBSAN236p35 genes	60%	CaMV35S (pEPT8)	[70]
*P. edulis*	Leave disks	*A. tumefaciens*	NIb and CP genes		pBluescript KS + vector	[71]
*P. edulis*	Leaf disks	*A. tumefaciens*	PWV CP gene	0.11–021	pCAMBIA 2300 binary vector	[72]
*P. edulis* var. *flavicarpa*	Leave disks	*A. tumefaciens*	CABMV-CP gene	0.19–0.0.67%	pCambia 2300 binary vector	[73]
*P. alat*	Hypocotyl	*A. tumefaciens*	pCABMV-dsCP-gene	0.89		[74]
*P. cincinnata* Mast.	Anther	*A. tumefaciens*	ß-Glucuronidase (uidA and nopaline synthase transcription terminator tNOS	57.89	pCAMBIA1304 (12,361 bp) binary vector	[76]
*P. edulis*	Wounded stem	*A. tumefaciens*	β-Glucuronidase (GUS), green fluorescent protein (GFP)	29%	pCAMBIA1301 and pCAMBIA1302	[78]
*P. cincinnata* and *P. edulis* f. *flavicarpa*	Root	*A. rhizogenes*	Chimaeric gene (nos-nptII-nos)	0.29	pRiA4b	[80]

There have been reports of transgenic *P. cincinnata* plants made from embryogenic cultures using sonication-aided *Agrobacterium*-mediated transformation (Table 2). The efficiency of passion fruit transformation may be improved if this approach is applied to *P. edulis* [5] Through *A. tumefaciens*-mediated transformation, transgenic *P. alata* lines containing a CABMV-derived CP gene fragment in a hairpin configuration were created [74] (Table 2). The response to CABMV infection was analyzed in twenty-one transgenic lines that had already been propagated. All transgenic lines had at least one propagated clone that was CABMV-infected after 4 consecutive mechanical inoculations, whereas 20 propagated clones from various transgenic lines remained healthy. Using RT-PCR to analyze these asymptomatic plants, CABMV was found in 17 of them, with consistently low virus titres compared to non-transgenic inoculation controls [74].

Through *A. tumefaciens*-mediated transformation, transgenic *P. alata* lines containing a CABMV-derived CP gene fragment in a hairpin configuration were created [74] (Table 2). The response to CABMV infection was assessed in twenty-one transgenic lines that had been propagated. All transgenic lines had at least one propagated clone that was CABMV-infected after four consecutive mechanical inoculations, while 20 propagated clones from various transgenic lines remained asymptomatic. Using RT-PCR to analyze these asymptomatic plants, CABMV was found in 17 of them, with consistently low virus titres compared to non-transgenic inoculation controls [74]. Genes that provide resistance to *X. axonopodis* pv. passiflorae and viral (CABMV) infections have been introduced to Passiflora species.

*Agrobacterium*-mediated transformation in passion fruit has been studied previously. However, it has not matured as transformation in other crops, such as pineapple [75], due to the long duration requirement and low transformation efficiency [76]. In addition, the *Agrobacterium*-mediated transformation efficiency in passion fruit was very low, ranging from 0.89 to 5.7% [74, 76]. Furthermore, there is no efficient transformation protocol for *Agrobacterium*-mediated transformation and gene function in Passiflora. In addition, several techniques, such as GUS gene transformation in leaf discs, transfer novel genes into passion fruit to examine their localization and function. Methods such as biolistic or particle gun bombardment and vector-mediated gene transformation by *A. tumefaciens* have been reported by several researchers [74, 77]. The successes of these transformations depended on several factors, such as the use of a proper selectable marker gene and suitable promoter with the sensitive selection agent, the use of suitable tissue or organ at the suitable developmental stage, and the availability of reproducible regeneration protocol. Moreover, there were earlier developed methods to study the passion fruit transformation, but they were time-consuming and less effective. In a recent study, Rizwan et al. [78] developed a new system of *Agrobacterium*-mediated genetic transformation through the cutting stem of seedlings which shows a high transformation percentage of about 29% (Table 2).

## Somatic hybridization

Somatic hybridization can develop novel rootstocks resistant to *Phytophthora* and *Fusarium*-caused soil-borne diseases. Somatic hybrids were created by [73] to introduce characteristics into passion fruit. Resistance to *Xanthomonas campestris* pv. *passiflorae* and fusarium wilt in *P. alata* and *P. cincinnata*, respectively. Other desirable features, including cold tolerance, have been addressed through the somatic hybridization of *P. incarnata* and *P. edulis* f. *flavicarpa* [79]. Protoplasm fusion and somatic hybridization could create new passion fruit in various forms, colors, and diameters [68, 80]. Established protoplast-to-plant regeneration systems for various passion fruit species and novel interspecies somatic hybrids have been generated among commercially yellow passion fruit and multiple wild relatives. Despite the diversity of *Passiflora* species and the method's relative simplicity, it has not been used to its maximum potential [113].

Passiflora’s protoplast isolation is impacted by several variables, including the genotype of the plant, the source tissue’s physiological condition, and environmental influences. Manders et al. invented protoplast isolation research in Passiflora. Since then, *P. edulis*, *P. gibertii*, *P. amethystina*, *P. cincinnata*, *P. coccinea*, and *P. incarnata* have all been found to regenerate from Passiflora protoplasts [79, 81–83]. Similar enzyme mixes, media based on Kao and Michayluk’s formulation, and similar culture methods, such as embedding protoplasts in fine layers or droplets of agarose-solidified media, have been employed in the majority of these experiments.

The novel hybridization of somatic hybrids has been established between numerous wild Passiflora species and cultivated yellow passion fruit [79, 81, 84, 85]. The somatic hybrid allotetraploids of *P. edulis* and *P. incarnata*, *P. edulis* and *P. cincinnata*, *P. edulis* and *P. alata*, *P. edulis* and *P. amethystina*, *P. edulis* and *P. gibertii*, and *P. edulis* and *P. cincinnata* have all been discovered [85]. The protoplast fusion of the frost-intolerant yellow passion fruit with the frost-tolerant wild *P. incarnata* was described by [86]. Four somatic hybrids reported by [87] suggested a phytoconstituents profile analysis that could reveal patterns of inheritance and synthesis of flavonoids [87]. Isolated from the leaves of *P. edulis*, *P. incarnata*, and their somatic hybrids were examined for flavonoids. *P. edulis* was discovered to contain isoorientin, whereas *P. incarnata* included vitexin. The flavonoid banding characteristics of all the somatic hybrids were comparable. There have been isoorientin and vitexin found in the somatic hybrids. The progenitor species’ high-performance liquid chromatography (HPLC) findings demonstrate a unique pattern of flavonoids. *P. edulis* had isoorientin that could be seen, although both species have isovitexin [17, 87].

Yellow passion fruit and *P. amethystina* hybrids created by [81] were matured and monitored in the field [84]. To introduce genes such as resistance to *X. campestris* pv. passiflorae from *P. cincinnata*, the hybrid showed steady meiotic behavior and normal pollen viability [84, 88]. The somatic hybrid *P. edulis* + *P. cincinnata* were described by [88]; however, recovered somatic hybrids were not acclimated and did not mature.

## Passion fruit polyploidy production

Breeders of wild passion fruit have employed various techniques to achieve polyploids [89]. Due to their relatively low fertility, polyploid individuals demonstrate more robust vegetative and reproductive vitality than their diploid relatives, with notably larger floral organs [90].

Some allotetraploids have already been confirmed for *Passiflora* species from traditional hybridization: (*P. eduli*s f. *flavicarpa* × *P. edulis* f. *sedulis*) × *P. incarnata*), P. ‘Byte’ (*P. edulis* × *P. incarnata*) × (*P. incarnata* × *P. cincinnata*), P. ‘Clear Sky’ (*P. amethystina* × *P. caerulea*) × *P. caerulea*) × *P. caerulea*), P. ‘Fertility’ (*P. incarnata* × *P. cincinnata*), *P. inspiration*’ (*P. incarnata* × *P. cincinnata*), P. ‘Ivy Waves’ (*P. coriacea* × *P. suberosa*), P. ‘Jara’ (*P. caerulea* × P. ‘Purple Haze’), P. ‘Manapany’ [(*P. edulis* × *P. incarnata*) × (*P. incarnata* × *P. cincinnata*)], P. ‘New Amethyst’ (*P. kermesina* × *P. caerulea*), and P. temptation’ (*P. incarnata* × *P. cincinnata*) [81, 91].

The culture of endosperm tissues has indeed been considered a direct technique for polyploid creation due to the triploid character of the endosperm. Mohamed et al. [92] were the first to use in-vitro endosperm culture to generate triploid *P. foetida* plants in *Passiflora*. With a mean of 1.9 shoots per each explant in basal medium with 2 μM BAP and 5 μM NAA, the authors reported the formation of shoots by direct somatic embryogenesis. *P. foetida* plants propagated from endosperms were found to be triploid. In contrast to diploid plants, triploid *P. foetida* plants had more vegetative vigor and larger leaves and flowers. *P. edulis* endosperm tissue can generate triploid and genetically stable plants [61]. The highest number of shoots were formed when endosperms were cultured on MS media supplemented with 9.0 μM TDZ. In addition, [61] also reported the plant growth from *P. cristalina* endosperm cultured on a medium containing 8.87 μM BAP, and the ploidy ratio of plantlets was not reported.

## Cryopreservation of *Passiflora* species

Protecting biodiversity, or the genetic diversity and diversity of life on the planet, is paramount for the future and the present. In breeding programs and for the establishment of new cultivars, conserving the variety at both the genetic and ecological levels is essential. Many plant species, wild varieties, and regional ornamental and fruit plants are presently on the edge of extinction. The most suitable technique for the long-term preservation of plant genetic materials is cryopreservation, which consists of storing tissue material in cylinders with liquid nitrogen (LN). However, creating an effective cryogenic system is a challenging endeavor that calls for considering several elements. It is particularly interesting to know how cryopreservation affects the stability and uniformity of the samples that have been stored [93]. Conservation and in-vitro cryopreservation are premised on plant propagation. They are complementary to traditional conservation practices for certain species and are the only feasible alternative for tropical and subtropical plants. These methodologies produced disease-free seed production with a high multiplication rate, lowering isolation necessities and enhancing germplasm exchange under controlled and axenic environments. Cell culture is also crucial for developing transgenic plants [6, 73] and high-value phytochemicals [93, 94].

Cryopreservation is the storage of biological material at extremely low temperatures, usually in liquid nitrogen (− 196 °C) or its vapor phase (− 150 °C), in a limited amount of space, protected them from contamination, and with little maintenance. All cellular divisions and metabolic events cease at this temperature, minimizing the chances of genetic modification [95]. As an outcome, it is considered the only available technique for maintaining safe and cost-effective long-term plant germplasm preservation. The first plant cryopreservation method was developed in the 1960s, based on the established mammalian cells technology. Since then, various protocols for other plant sources of thousands of species have been developed (Table 3).

**Table 3:** In vitro conservation of *Passiflora* spp.

Evaluated species	Aim of conservation	Preserved organ	Cryopreservation technique	References
*P. pohlii* Mast.	To develop a cryopreservation technique for the root system by using the V-Cryo-plate technique and characterize the anatomical alterations that occurred during the protocol's consecutive stage	Root	V-Cryo-plate technique	[101]
*P. suberosa* L.	To study the influence of explant age and exposure to the vitrification solutions PVS2 and PVS3 being evaluated. Furthermore, the occurrence of oxidative stress was analyzed at various phases of the protocol by measuring oxidative damage and the antioxidant defense enzymes	Shoot tip	V-Cryo-plate technique	[102]
*P. pinnatistipula*, *P. tarminiana*, *P. mollissima*	To develop cryopreservation protocols for recalcitrant or intermediate seeds which provide a viable method of long-term germplasm conservation	Seed	Encapsulation-cryopreservation technique	[104]
*P. alata*, *P. cincinnata*, *P. coriacea*, *P. edulis*, *P. edulis* f. *flavicarpa*, *P. foetida*, *P. giberti*, *P. micropetala*, *P. morifolia*, *P. nitida*)	To test germination percentage of 10 species	Seed	Liquid nitrogen (− 196 °C)	[107]
*P. pohlii*	To the development of in vitro preservation using axenic plant nodal segments	Nodal segments	Encapsulation-vitrification and vitrification techniques	[108]
*P. edulis*	to achieve the efficient cryopreservation of plant embryos by providing their rapid (5 min), uniform permeation by Plant Vitrification Solution cryoprotectant	Zygotic embryo	Vacuum infiltration vitrification (VIV),	[109]
*P. eichleriana* Mast., *P. nitida* Kunth., *P. mucronata* Lam.	To examine X-rays to check for any damage brought over by cryopreservation or defrosting	Seed	Liquid nitrogen (− 196 °C)	[111]

It cannot be overstated how important it is to collect and preserve the variety of *Passiflora* germplasm, both as a source of genes and natural products and for its biological importance [96]. Ex situ conservation strategies have generally been used to conserve *Passiflora* genetic resources in germplasm banks [13, 97, 98]. At the same time, periodical renewal is restricted by the decline in germination ability, which causes material damage. In vitro conservation programs for the species, seed preservation [99, 100], and cryopreservation of shoot and root tips by the V-Cryo-plate technique [101, 102] have been the focus of numerous research organizations [96, 103]. Seed and in vitro propagules of passion fruit species have been preserved via cryopreservation. Since collecting seeds from wild populations is challenging due to habitat degradation, research has been focused on the cryopreservation of shoots and nodal fragments. The most basic methods are cryopreservation and encapsulation-vitrification. The cryopreservation was employed for *P. tarminiana*, *P. pinnatistipula*, and *P. mollissima*. However, only *P. foetida* shoot tips and seeds had a post-freeze recovery of 60% when cryopreserved via the encapsulation-vitrification technique [104–106]. Seed cryopreservation of 10 *Passiflora* species in LN showed that the final germination percentage was not affected in LN [105, 107].

Cryopreservation of nodal segments might not be the best option. However, utilizing the vitrification technology, a promising cryopreservation method for nodal segments of *P. pohlii *in vitro plants was devised [108]. Employing the encapsulation-vitrification method, the shoot tips of *P. suberosa* had the best survival rates (28%) upon pre-treatment with 0.3 M sucrose for 24 h, exposition to PVS2 for 60 min, and post-freezing incubation in the dark for 60 days in MSM basal medium with 0.44 μM BAP [58]. Similar data were shown with *P. foetida* shoot tips, which only revealed post-freezing recovery (60%) when the encapsulation-vitrification technique was employed [96]. The type of vitrification solution had a major effect on recoveries in both species since PVS2 exposure led to greater recovery rates over PVS3 exposure, which is generally composed of being less cytotoxic due to its size and differentiated condition. However, [108] successfully developed a vitrification-based cryopreservation methodology for nodal segments of *P. pohlii *in vitro plants. After pre-growing on MSM medium supplemented with 0.7 M sucrose and being exposed to PVS3 for 30–120 min before immersion in LN, the best recovery rate (65%) was noted. Recovery was achieved using MSM media supplemented with 30.8 μM BAP and maintained for 30 days in the dark before being subjected to light. Recently, a novel method of cryopreserving *P. edulis* zygotic embryos was reported [109]. Vacuum infiltration vitrification (VIV), a variation of this technique, guarantees quick and uniform absorption of PVS2 and good post-thaw recovery [110]. Considering recent progress, prospects include improving the existing cryopreservation techniques and developing new ones. To examine damage causedd by cryopreservation or defrosting, X-rays are used to check for any damage (Table 3) [111].

## Conclusion

For several passion fruit research groups worldwide, inefficient seedlings regeneration from in vitro culture methods remains an important barrier. This is related to unanswered or poorly resolved issues with the inconsistent response of callus-derived tissues, delayed growth of in vitro tissues, and their resulting lack of vigor when planted ex vitro. Due to these factors, passionfruit breeding has experienced less progress than many other plant species. To promote future progress in passionfruit micropropagation, it is vital to consider a wise step and then apply techniques that have been programmed to achieve highly efficient in vitro regeneration. According to the literature, it could create extremely effective embryogenic cell suspension cultures from chosen callus lines to overcome current difficulties and create a quick clonal propagation method for passion fruit. As a result, future studies should concentrate on improving in vitro circumstances by employing medium supplements and cell suspension culture technology to boost the induction of somatic embryos. With brief immersion and photoautotrophic systems, subsequent development and acclimation might be further enhanced. Further research may reveal the role of media composition in encouraging somatic germination in *Passiflora* species. Besides, the use of molecular methods to detect the genes responsible for controlling somatic embryogenesis may increase the rate of somatic embryogenesis.

However, genetic transformation via leave, anther, and cotyledon transformation was established for several *Passiflora* species, but still, further research through indirect somatic embryo regeneration is required.

New molecular techniques like CRISPR/Cas9 soon be available to investigate further gene function and modification. The advancement of CRISPR/Cas9 technologies has opened up new possibilities for the genome editing of several plants. CRISPR/Cas9 is a genome editing method for site-direct mutagenesis that contains several advantageous properties that are not possible with conventional mutagenic techniques, such as efficiency, specificity, low cost, and simplicity of application. It could also be used to construct mutant libraries. CRISPR/Cas9 for passionfruit breeding, particularly concerning yield, quality, abiotic and biotic stresses resistance traits, has gained favor among researchers studying other plant genomics. CRISPR/Cas9 is alluring because it can develop genome-edited, transgene-free plants. So far, it indicates that CRISPR/Cas9 system is useful for improving plant breeding. However, the difficulties and worries associated with the widespread use of the CRISPR/Cas9 system could impede its further development.

## Data Availability

Not applicable.
